# Enhanced xylitol production using non-detoxified xylose rich pre-hydrolysate from sugarcane bagasse by newly isolated *Pichia fermentans*

**DOI:** 10.1186/s13068-020-01845-2

**Published:** 2020-12-29

**Authors:** Ashish A. Prabhu, Ekkarin Bosakornranut, Yassin Amraoui, Deepti Agrawal, Frederic Coulon, Vivekanand Vivekanand, Vijay Kumar Thakur, Vinod Kumar

**Affiliations:** 1 School of Water, Energy and Environment, Cranfield University, Cranfield, MK43 0AL UK; 2 Biochemistry and Biotechnology Area, Material Resource Efficiency Division, CSIR-Indian Institute of Petroleum, Mohkampur, Dehradun, 248005 India; 3 Centre for Energy and Environment, Malaviya National Institute of Technology, Jaipur, Rajasthan 302017 India; 4 Biorefining and Advanced Materials Research Centre, Scotland’s Rural College (SRUC), Edinburgh, UK

**Keywords:** Xylose, Sugarcane bagasse pre-hydrolysate, *Pichia fermentans*, Xylitol, Chemical mutagenesis

## Abstract

**Background:**

Integrated management of hemicellulosic fraction and its economical transformation to value-added products is the key driver towards sustainable lignocellulosic biorefineries. In this aspect, microbial cell factories are harnessed for the sustainable production of commercially viable biochemicals by valorising C5 and C6 sugars generated from agro-industrial waste. However, in the terrestrial ecosystem, microbial systems can efficiently consume glucose. On the contrary, pentose sugars are less preferred carbon source as most of the microbes lack metabolic pathway for their utilization. The effective utilization of both pentose and hexose sugars is key for economical biorefinery.

**Results:**

Bioprospecting the food waste and selective enrichment on xylose-rich medium led to screening and isolation of yeast which was phylogenetically identified as *Pichia fermentans*. The newly isolated xylose assimilating yeast was explored for xylitol production. The wild type strain robustly grew on xylose and produced xylitol with > 40% conversion yield. Chemical mutagenesis of isolated yeast with ethyl methanesulphonate (EMS) yielded seven mutants. The mutant obtained after 15 min EMS exposure, exhibited best xylose bioconversion efficiency. This mutant under shake flask conditions produced maximum xylitol titer and yield of 34.0 g/L and 0.68 g/g, respectively. However, under the same conditions, the control wild type strain accumulated 27.0 g/L xylitol with a conversion yield of 0.45 g/g. Improved performance of the mutant was attributed to 34.6% activity enhancement in xylose reductase with simultaneous reduction of xylitol dehydrogenase activity by 22.9%. Later, the culture medium was optimized using statistical design and validated at shake flask and bioreactor level. Bioreactor studies affirmed the competence of the mutant for xylitol accumulation. The xylitol titer and yield obtained with pure xylose were 98.9 g/L and 0.67 g/g, respectively. In comparison, xylitol produced using non-detoxified xylose rich pre-hydrolysate from sugarcane bagasse was 79.0 g/L with an overall yield of 0.54 g/g.

**Conclusion:**

This study demonstrates the potential of newly isolated *P. fermentans* in successfully valorising the hemicellulosic fraction for the sustainable xylitol production.

## Background

In the present global scenario, tremendous efforts are being made in search of the alternative source to overcome the reliance on petroleum products. The economy of a biorefineries primarily  thrives on successful extraction of renewable carbon present in the biomass in terrestrial biosphere and its transformation to economically viable products such as chemicals, fuels, energy, polymer etc. [1]. Lignocellulosic biomass (LCB) is the significant source of fixed organic carbon which is not only renewable but cheap and abundant in nature. LCB principally consists of three components; cellulose, hemicellulose and lignin. As opposed to lignin which is a complex heterogeneous polymer, cellulosic and hemicellulosic fractions are more lucrative options for valorization. These structural polysaccharides form the largest fraction of the total biomass and provide a sugar platform upon depolymerization, which can be later transformed into a range of commercially important bio-renewable products [2, 3]. In this steer, the UK government has shown a great interest in the efficient utilization of LCB to foster the bio-economy. In 2014, Lignocellulosic Biorefinery Network (LBNet) was commissioned to identify the current availability of the LCB in the UK, explore its potential and expand its utility for the development of integrated biorefinery. The analysis highlighted that over 16 million tons of biomass, including green waste and agricultural straw, are generated per year in UK [4]. The remit of LBNet has been broadened with a new name as BBNet (Biomass and Biorefinery Network) with the aim to build a dynamic community of industrial and academic partners, to develop sustainable routes for the transformation of non-food biomass into fuels, chemicals and materials. The government bodies such as Biotechnology and Biological Sciences Research Council (BBSRC) is empowering the industrial biotechnology and bioenergy concept and bringing the industry-academia conglomeration to convert renewable raw material to value-added products [5].

However, state of the art reveals that presently most of the research is focused towards depolymerization  of the cellulosic fraction and its commercial exploitation. LCB-based biorefineries can simultaneously achieve the target of enhanced carbon efficiency, techno-commercial success and waste minimisation if the potential of hemicellulose is also unleashed simultaneously [2, 3]. Hemicellulose is a branched hetero-polysaccharide, composed mainly of pentose monomeric sugars specifically xylose which makes up to 30–40% of LCB [6]. Xylose can be valorized  into an array of industrially important chemicals such as furfural, xylitol, ethanol, lactic acid, propionic acid, butyric acid, biopolymers, etc. either through chemical or biotechnological route [1]. However, the xylose valorization  through biochemical route is largely ignored, as most of the industrial microbes lack efficient metabolic pathway for its assimilation. More eminently, xylose utilization in bacteria, fungi and yeast suffers a major setback due to the preference of microbes for glucose as carbon substrate over xylose, commonly known as carbon catabolite repression [7].

Selective fractionation of hemicellulose and its hydrolysis is possible only when the pretreatment of LCB is done via dilute acid (DA) or hydrothermal treatment. Both these popular pretreatment strategies generate pre-hydrolysate rich in C5 sugars and cellulolignin biomass. Use of dilute acid reduces the process severity (≥ 120 °C), but during hydrothermal pretreatment, very high temperatures (≥ 180 °C) are required for water to act as Lewis acid. The intensity of process conditions, the residence time of the biomass and the concentration of acid in DA pretreatment control the generation of lignocellulose-derived by-products and their quantities [8, 9]. Disruption of lignin–carbohydrate complex (LCC) linkages during such pretreatment invariably releases fermentation inhibitors such as weak acids, furan derivatives, phenolics etc. which suppress the microbial growth during fermentation step. To counteract this problem generally an additional step of pre-hydrolysate detoxification is incorporated. Therefore, detoxification methods such as over liming, activated charcoal, ion-exchange resin, adsorption or a combination of these methods is required to remove these inhibitors for a smooth and effective fermentation. The process of detoxification need additional facilities and significantly increases the capital investment. Moreover, this step usually leads to sugar loss, reducing fermentation performance and product yields [10–12]. Alternatively, microbial strains should be resistant to these toxic inhibitors making it more worthwhile for cheap fermentations.

Among all the xylose derived value-added chemicals, xylitol production through biotechnological intervention has been one of the most studied products. Unlike conventional chemical route, xylitol production via microbial pathway is less energy-intensive, environmentally benign and offers numerous advantages such as reduced purification cost of xylose and direct use of biomass-derived mixed sugars [13, 14]. Xylitol is recognised as one of the 12 most promising platform chemicals, owing to its transformation potential into value-added products such as xylaric acid, glycerol, lactic acid, ethylene glycol, propylene glycol and derivatives of hydroxyfurans. Further, the low glycemic index of xylitol and its antimicrobial activity against gram-positive bacteria makes it an essential ingredient of many food products. Since xylitol finds significant  applications in food and pharmaceuticals, the microbial fermentation of xylose to xylitol should preferably make use of an organism with GRAS (generally regarded as safe) status [13, 15, 16].

In this context, the present study aimed towards exploring the xylitol producing potential of the newly isolated xylose assimilating yeast isolated from food waste. Molecular characterization  confirmed that the said isolate belonged to the genus “*Pichia*” or most commonly termed as “*Scheffersomyces*” which is known to have a “GRAS” status [17, 18]. Strain improvement was attempted by chemical mutagenesis followed by statistical optimisation of culture medium using the best xylitol producing mutant strain. After optimization , the xylitol production studies were conducted both at shake flask and bioreactor levels. The capability of the said strain was evaluated with non-detoxified xylose rich pre-hydrolysate obtained after hydrothermal pretreatment of sugarcane bagasse (SCB) using pure xylose as the benchmark. To the best of our knowledge, this is the first report on xylose assimilation and xylitol production by *P. fermentans*.

## Results and discussion

### Shake flask studies for assessing xylitol production ability of *P. fermentans*

**Fig. 1.**
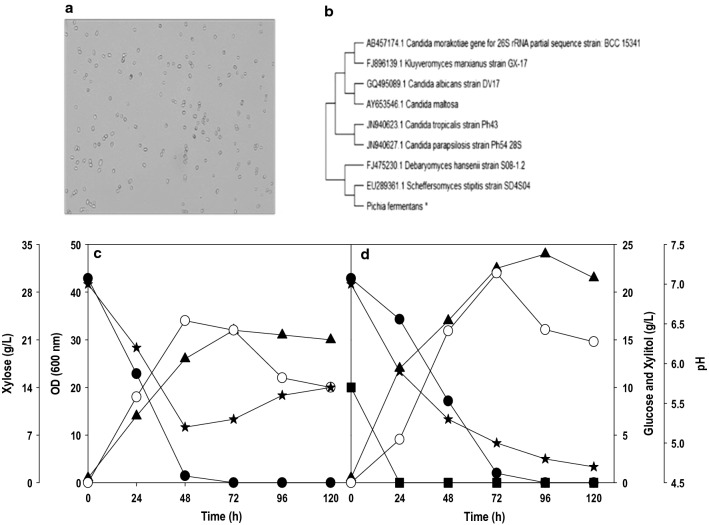
**a** Morphological identification of *Pichia fermentans *by microscopy. **b** Phylogenetic tree based on 26s rRNA sequence, using neighbor joining method clustered with bootstrap test with 1000 replicates. The isolated strain reported in the present study is marked with a star. Time-course profiles for shake flask cultivation of *P. fermentans *on; **c** xylose and **d** xylose + glucose. Symbols: filled square (glucose), filled circle (xylose), filled triangle (OD_600_), empty circle (xylitol) and filled star (pH). The graphs represent the average values of triplicate experiments performed with less than 10% S.D (standard deviation)

The isolated yeast strain was examined for its ability to assimilate xylose and further fermenting it to xylitol. When *P. fermentans* was grown on pure xylose, there was rapid consumption of xylose and nearly the entire xylose was depleted in 48 h with a concomitant rise in cell growth (Fig. 1c). The cell growth was rapid, and an OD_600_ of 14 was recorded in 24 h with a steady increase till it attained an absorbance of ≥ 30. The starting pH was 7.0 and dropped below 5.5 after 48 h. The xylitol accumulation of 9.0 g/L was noticed at 24 h and the highest concentration of 17 g/L was recorded at 48 h with a yield of 0.57 (g/g). These results show the strong ability of isolated *P. fermentans* to grow on xylose and valorize/ferment it to xylitol. The co-fermentation of glucose and xylose by *P. fermentans* was carried out and the impact of the presence of glucose on the growth pattern, substrate utilization and xylitol formation was investigated (Fig. 1d). Glucose was preferred over xylose which led to the rapid depletion of glucose, and the initial glucose (10.0 g/L) was consumed within 24 h of fermentation. The xylose consumption was slowed down in the presence of glucose and rapidly enhanced after glucose exhaustion in the next 48 h. The addition of glucose boosted up cell growth, and significantly, higher OD_600_ was obtained. As a result of it, more xylose was diverted towards xylitol formation during co-fermentation than fermentation with only xylose. By 72 h, ~ 95% of the xylose was consumed, and the maximum of 22 g/L xylitol was produced with conversion yield being 0.77 g/g. A small amount of glucose is helpful for overall xylitol accumulation, and higher xylitol yield in mixed sugar fermentation could be attributed to rapid build-up of biomass from fast glucose depletion [19]. The suppressed utilization of other carbon sources in the presence of glucose is quite common. It could be attributed either to carbon repression enzymes, which suppress the xylose uptake or competition between the transport carriers as most of the yeast transport system shows high affinity toward hexose sugars and low affinity for pentose sugars [1, 20]. The results obtained can be correlated to a similar study by Oh and Kim, which unveiled fast glucose consumption in mixture sugar fermentation and high xylitol production by *C. tropicalis* [21].

The biological production of xylitol has been investigated for a long time. Though the variety of organisms including bacteria, yeasts and fungi have been explored for xylitol accumulation, yeasts such as *Candida, Hansunela, Debaromyces, Spathaspora, Pachysolen, Kluyveromyces, Pichia* etc. are the most promising xylitol producers. Among yeasts, *Candida *sp. such as *C. boidinii*, *C. guillermondii*, *C. tropicalis* etc. are the most extensively studied organisms [13–15]. However, this genus is a known opportunistic pathogen and associated with a number of skin disorders. Xylitol finds major applications in food-based industries and pathogenicity of *Candida* sp. impedes the commercial applications of the yeast. On the other hand, *P. fermentans *is a non-conventional, non-pathogenic, safe, dimorphic and fast-growing yeast even under microaerophilic conditions. The yeast produces flavour and aroma compounds which find wide applications in food and beverage industries. *P. fermentans* has antagonistic properties which tends to inhibit the growth of fungi and bacteria, and thereby, enhances the shelf life of food and beverage products [22, 23], making it an attractive candidate for the xylitol production.

### Chemical-based mutagenesis of *P. fermentans*

The random mutagenesis is unpredictable compared to rational design and can significantly alter the genome of the microbes creating a strain having superior phenotypic characteristics.

**Fig. 2.**
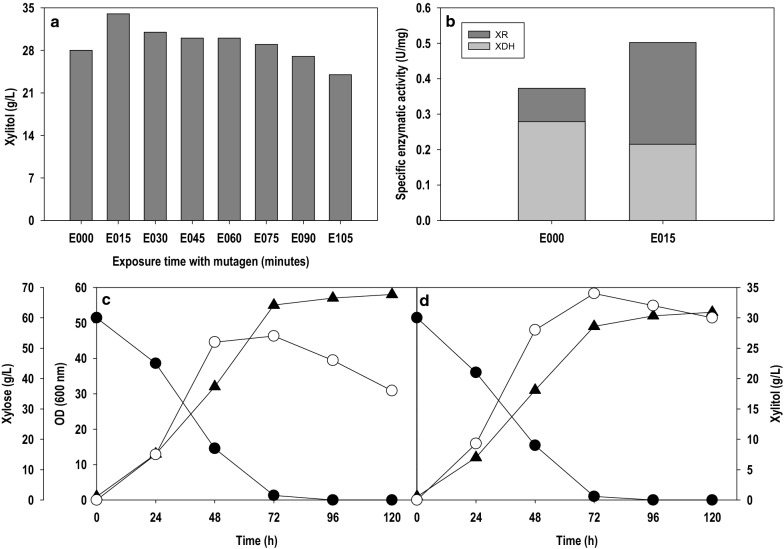
**a** Exposure of wild type *P. fermentans *with chemical mutagen EMS for different times; **b** Specific enzymatic activity of xylose reductase and xylitol dehydrogenase during fermentation; Batch fermentation for xylitol production by **c** wild type and **d** mutant (E015) strain of *P. fermentans*. Symbols: filled circle (xylose), filled triangle (OD_600_) and empty circle (xylitol). The graphs represent the average values of triplicate experiments performed with less than 10% S.D

Microbial systems with desired traits can be obtained by the application of inverse metabolic engineering, which causes random mutations in the genome [24]. In the present study, *P. fermentans* was subjected to chemical mutagenesis by exposing the growing cells to 20 mM ethyl methanesulphonate (EMS) for different time intervals. When all the mutants were screened for their ability to produce xylitol with parent strain as the yardstick, xylitol accumulated was equal or more than the parent strain for an exposure time up to 90 min and declined thereafter as shown in Fig. 2a. The best results were obtained with the mutant strain, which was exposed to EMS for 15 min (E015). Figure 2c, d shows batch fermentations of wild type (E000) and mutant (E015) strains in shake flask. The batch cultivations were carried out with initial xylose level of 60.0 g/L. Nearly all the xylose was consumed by both the strains within 72 h time duration and exhibited similar cell growth patterns (OD_600_: 52–58). However, a significant difference was recorded in terms of xylitol production. The mutant strain (E015) produced the maximum xylitol titer and yield of 34 g/L and 0.68 g/g, respectively, 26% higher in comparison to wild type strain (E000) which accumulated 27 g/L xylitol with a conversion yield of 0.45 g/g. Our results are comparable to Kim et al. 2015 [24], where they have subjected the *K. marxianus* ATCC 36907 to EMS based mutagenesis and reported 1.79 fold increase in the xylitol titer (25 g/L) in comparison to the parent strain (14 g/L).

The biosynthesis of xylitol is a single-step reduction reaction and tightly linked to the availability of reduced cofactors. The reaction involves the reduction of xylose using electrons from redox co-factor NAD(P)H and is catalysed by xylose reductase (XR). Therefore, continuous replenishment of reduced cofactor is required for smooth production of xylitol. In the next step, xylitol is oxidized to xylulose via NAD^+^ and the reaction is mediated through xylitol dehydrogenase (XDH) [25]. The xylitol formed is partially excreted and partially metabolized to xylulose, which subsequently enters central carbon metabolism for biosynthesis of precursors and eventually contributes to cellular growth. Thus, activities of XR and XDH and availability of redox co-factors play an essential role in determining xylitol level.

In this study, the enzymatic activities of XR and XDH were quantified to unravel the superior performance of E015 over its parent strain E000. Figure 2b compares the activities of these two enzymes in wild type and mutant (E015) strains. The enzymatic activity was carried out from the 48 h old cultures when the culture growth was in the mid-exponential phase, and most of the xylose was consumed. EMS treatment caused a significant change in XR and XDH activity levels of the wild type strain. In E015 mutant, the XR activity was enhanced by 34.6% while XDH activity was decreased by 22.9%. As a result, the XR/XDH ratio changed from 1.34 in wild type to 2.33 in the mutant strain, which was reflected in the xylitol production. On the other hand, the better synchronisation of XR and XDH in wild type strain due to narrow gap in their activities probably allowed more carbon flux towards central carbon metabolism and resulted in lower xylitol accumulation. It was observed that xylitol concentration dropped after reaching the peak when xylose was exhausted, indicating xylitol serving as a carbon source in the absence of xylose and re-entering the metabolism. An increase in cell OD_600_ also supported this observation. However, the drop was more rapid in wild type than the mutant strain, which can be correlated with low XDH activity of mutant strain. Balanced XR: XDH ratio plays a crucial role in xylitol formation. The observation made in the current work is consistent with the literature information. For example, Gírio et al. [26] achieved high xylitol production when XR activity was almost double of XDH in *Debaryomyces hansenii*. Walfridsson et al. [27] reported that *Pichia stipitis* strain exhibiting XR/XDH activity ratio of 17.5 produced 0.82 g xylitol/g xylose while observed no xylitol with the yeast strain having low XR/XDH ratio of 0.06.

### Xylitol production from non-detoxified xylose rich lignocellulosic pre-hydrolysate

**Fig. 3.**
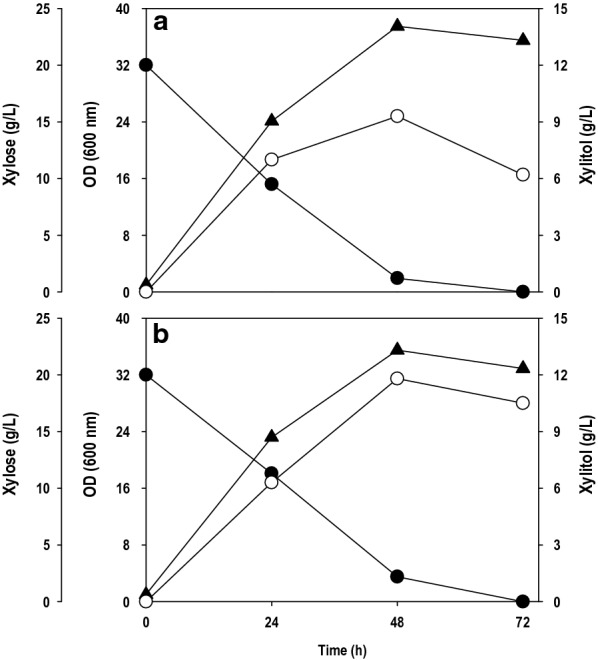
Xylitol production using xylose rich hemicellulosic pre-hydrolysate from sugarcane bagasse by **a** wild type and **b** mutant strain of *P. fermentans*. Symbols: filled circle (xylose), filled triangle (OD600) and empty circle (xylitol). The graphs represent the average values of triplicate experiments performed with less than 10% S.D

Agriculture wastes such as corn cobs, rice straw, SCB, wood chips etc. contains a considerable amount of hemicellulose content and are cheaper feedstock in comparison to pure xylose [1, 3]. Despite all the cited drawbacks, presently microbial platforms using biomass-derived xylose are predominated by yeasts owing to their robust nature and high tolerance to inhibitory compounds associated with it. Therefore, it was imperative to check the robustness of *P. fermentans* to manufacture xylitol using hemicellulosic feedstocks. To this end, the xylitol production ability of *P. fermentans* was investigated at shake flask level, using non-detoxified xylose rich pre-hydrolysate obtained by hydrothermal pretreatment of SCB. The toxic effect of acetic acid was extenuated by adjusting the pH of pre-hydrolysate to 7.0 [28]. The wild type (E000) and mutant (E015) *P. fermentans* strains were grown on pre-hydrolysate containing 20.0 g/L xylose (Fig. 3a, b). The xylose consumption was smooth as with pure xylose, and a large fraction of initial xylose concentration of 20.0 g/L was consumed within 48 h. Both the strains exhibited high cell growth (OD_600_: 35–38) concomitant with xylose assimilation. The xylitol titer and yield obtained with mutant strain were 11.8 g/L and 0.59 g/g, respectively, while wild type strain was able to accumulate 9.3 g/L xylitol with a conversion yield of 0.46 g/g. Again, mutant outperformed wild type strain for xylitol production and, therefore, was used in further experiments. Despite the presence of the substantial amount of acetic acid and little furfural in pre-hydrolysate, the strain grew very well and accumulated a significant amount of xylitol. The high cell concentration in inoculum used and cell density achieved during fermentation might be favourable for mitigating toxic effects of inhibitors [29]. The results obtained in terms of xylose metabolism, cell growth and product yield were similar to pure xylose indicating the robustness of *P. fermentans* in valorizing  lignocellulosic feedstock.

### Effect of aeration on xylitol production in shake flask

Aeration plays a game-changing role in xylitol production, especially in yeasts, as it affects the activities of XR and XDH, thereby regulating xylose metabolism [15, 30]. The partition of xylose flux between xylitol synthesis and cell growth is highly dependent on oxygen levels. Under high aeration condition, the NADH formed in the second step is re-oxidized favouring oxidation of xylitol to xylulose. As a result, the xylitol formed enters the pentose phosphate metabolism, which is subsequently metabolized to cell mass and other metabolites and resulting in less xylitol accumulation. Conversely, oxygen deprived/limited condition will result in increased intracellular NADH levels which leads to an imbalance between XR and XDH, causing xylitol accumulation [13, 25, 31]. Thus, it was indispensable to study the effect of aeration on the xylitol production by mutant strain E015, as it altered the dissolved oxygen and hence affected the oxygen uptake rate as well.

**Fig. 4.**
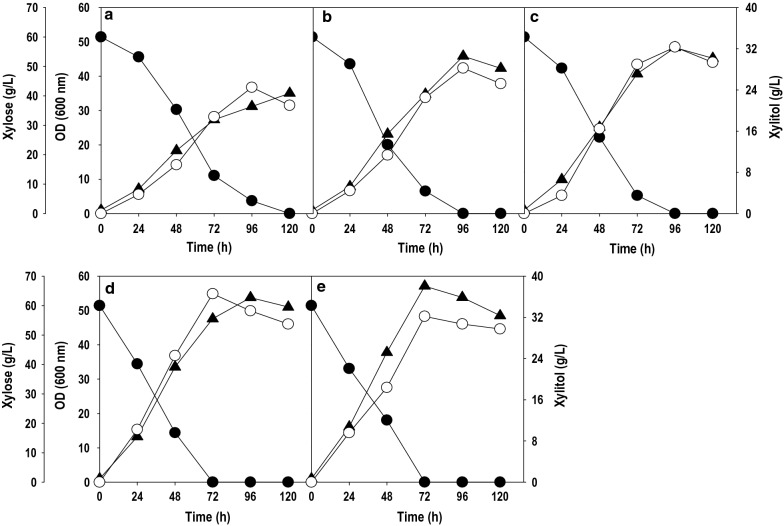
Impact of agitation speed on xylose uptake, cell growth and xylitol formation by *P. fermentans* in shake flask **a** 100 rpm, **b** 150 rpm, **c** 200 rpm, **d** 250 rpm and **e** 300 rpm. Symbols: filled circle (xylose), filled triangle (OD_600_) and empty circle (xylitol). The graphs represent the average values of triplicate experiments performed with less than 10% S.D

There are two ways to play with the oxygen levels at the shake flask level. The first method is by varying the agitation speed of the shaker and other being altering the media volume in a flask of fixed capacity. Figure 4 depicts the effect of aeration on xylitol production, where the flasks containing *P.fermentans* were grown   at 100 to 300 rpm with an interval of 50 rpm. Complete utilization of xylose occurred within 120 h at 100 rpm and the consumption time significantly reduced with increase in shaking speed. It was 96 h at 150 and 200 rpm while at 250 and 300 rpm, it was reduced to 72 h. The cell growth increased continuously as agitation speed was raised from 100 (OD_600_: 35.0) to 300 rpm (OD_600_: 57.1). The xylitol titer and yield also enhanced linearly with an increase in agitation till 250 rpm and thereafter, the numbers dropped. The highest titer and yield of 36.6 g/L and 0.61 g/g were recorded at 250 rpm. On the other hand, increasing agitation speed to 300 rpm resulted in higher biomass production but reduced xylitol accumulation.

The results of the present study are in agreement with literature which suggested that higher agitation speed enhanced cell growth while moderate agitation speed was found to be beneficial for xylitol accumulation. Xylose to xylitol conversion is a biotransformation reaction, and therefore high cell density fermentation is recommended for accumulating high product titers. However, a biomass concentration beyond a given threshold could impede xylitol biosynthesis by imposing oxygen limitation [32]. A similar observation was made by Unrean and Ketsub inferring that increment in oxygen levels up to certain level enhanced cell growth and xylitol production, and too high oxygen levels reduced xylitol accumulation [33]. Zhang et al. attempted both single-stage and two-stage fermentation in shake flask to investigate the impact of shaking speed on xylitol accumulation by *Candida athensensis* strain [34]. They achieved the highest xylitol concentration of 112.6 g/L with xylose to xylitol bioconversion efficiency of 82.6% in single stage fermentation at 150 rpm. In two-stage fermentation, 200 rpm was maintained for first 36 h and thereafter, agitation speed was reduced to 100 rpm for remaining time. This arrangement resulted in maximal xylitol production (115.6 g/L) and highest bioconversion efficiency of 84.6%. In both cases, cell growth was compromised to maximize xylitol formation. Thus, a trade-off between the biomass formation and xylitol accumulation requires a moderate shaking speed or a microaerobic condition to maximize the xylitol yield. All these results show a fine-tuning of oxygen level is necessary for the high-level accumulation of xylitol.

### Optimization of media components for maximizing xylitol production

To analyze the effect of medium components on xylitol production, we adopted a three-level Box Behnken design. The design matrix, along with the observed and predicted responses for xylitol production, is shown in Table 1. Multiple regression analysis was carried out to study the model accuracy and based on the evaluation, second-order polynomial model was fitted to the equation.

**Table 1:** Box Behnken design matrix with un-coded values along with observed and predicted response for xylitol production

Xylose (g/L)	Ammonium sulphate (g/L)	KH_2_PO_4_ (g/L)	Yeast extract (g/L)	Xylitol (g/L)(Exp)	Xylitol (g/L)(Pred)
50	0.1	0.55	11	14.4	14.39
150	0.1	0.55	11	32.33	31.91
50	1	0.55	11	10.17	11.59
150	1	0.55	11	21.02	22.04
100	0.55	0.1	2	20.53	20.10
100	0.55	1	2	17.81	17.28
100	0.55	0.1	20	19.05	20.58
100	0.55	1	20	12.65	14.08
50	0.55	0.55	2	32.48	32.76
150	0.55	0.55	2	25.96	27.79
50	0.55	0.55	20	14.04	12.44
150	0.55	0.55	20	45.42	45.38
100	0.1	0.1	11	11.52	13.82
100	1	0.1	11	7.93	7.64
100	0.1	1	11	8.78	9.31
100	1	1	11	4.89	2.83
50	0.55	0.1	11	11.03	10.05
150	0.55	0.1	11	44.89	42.75
50	0.55	1	11	23.21	24.10
150	0.55	1	11	19.64	19.38
100	0.1	0.55	2	18.03	16.87
100	1	0.55	2	12.33	12.33
100	0.1	0.55	20	18.55	17.31
100	1	0.55	20	9.26	9.18
100	0.55	0.55	11	34.17	35.05
100	0.55	0.55	11	36.04	35.05
100	0.55	0.55	11	37.85	35.05
100	0.55	0.55	11	34.22	35.05
100	0.55	0.55	11	32.99	35.05

*Exp* experimental, *Pred* predicted

$$ \begin{aligned}{Y}_{\mathrm{Xylitol}}\,=&\,35.05+{6.99X}_{1}-{3.16X}_{2}-{2.33X}_{3}\\ &-{0.68X}_{4}+0.30{X}_{1}^{2}-{15.37X}_{2}^{2}-11.28{X}_{3}^{2}\\ &-5.75{X}_{4}^{2}-1.77{X}_{1}{X}_{2}-9.35 {X}_{1}{X}_{3}+9.47{X}_{1}{X}_{4}\\ &-0.075{X}_{2}{X}_{3}-{0.89X}_{2}{X}_{4}-0.92{X}_{3}{X}_{4}\end{aligned} $$

where, *X*_1_ = xylose (g/L), *X*_2_ = ammonium sulphate (g/L), *X*_3_ = KH_2_PO_4_ (g/L) and *X*_4_ = yeast extract (g/L).

Table 2 shows the statistical tool ANOVA that was used to interpret the results. Fisher *F* test was highly significant, with *p* < 0.05. The coefficient of determination (*R*^2^) was used to determine the model’s goodness of fit and it was found to be 0.98, which leads to the conclusion that 98.0% of the variation in the model could be explained. Another function called the “lack of fit” analyses the failure of the model and represents data in experimental domains at points not mentioned in the model. Herein, the model lack of fit was found to be insignificant (*p* > 0.05) with *F* value of 0.92.

**Table 2:** ANOVA for quadratic model

Source	DF	Seq SS	Adj SS	Adj MS	*F* value	*p* value
Regression	14	3685.87	3685.87	263.28	77.1	0
Linear	4	778.17	778.17	194.54	56.97	0
X1	1	587.02	587.02	587.02	171.9	0
X2	1	120.4	120.4	120.4	35.26	0
X3	1	65.19	65.19	65.19	19.09	0.001
X4	1	5.56	5.56	5.56	1.63	0.223
Square	4	2179.18	2179.18	544.79	159.54	0
X1 * X1	1	158.66	0.58	0.58	0.17	0.685
X2 * X2	1	1106.41	1532.83	1532.83	448.87	0
X3 * X3	1	698.9	825.68	825.68	241.79	0
X4 * X4	1	215.2	215.2	215.2	63.02	0
Interaction	6	728.52	728.52	121.42	35.56	0
X1 * X2	1	12.53	12.53	12.53	3.67	0.076
X1 * X3	1	350.25	350.25	350.25	102.57	0
X1 * X4	1	359.1	359.1	359.1	105.16	0
X2 * X3	1	0.02	0.02	0.02	0.01	0.936
X2 * X4	1	3.22	3.22	3.22	0.94	0.348
X3 * X4	1	3.39	3.39	3.39	0.99	0.336
Residual error	14	47.81	47.81	3.41		
Lack-of-fit	10	33.28	33.28	3.33	0.92	0.588
Pure error	4	14.53	14.53	3.63		
Total	28	3733.67				

DF = Degree of freedom; Seq SS = sequential sum of square; Adj SS = adjusted sum of square; Adj MS = adjusted mean square; *F* = variance ratio (Fisher *F*-value); *p* = probability value

**Fig. 5.**
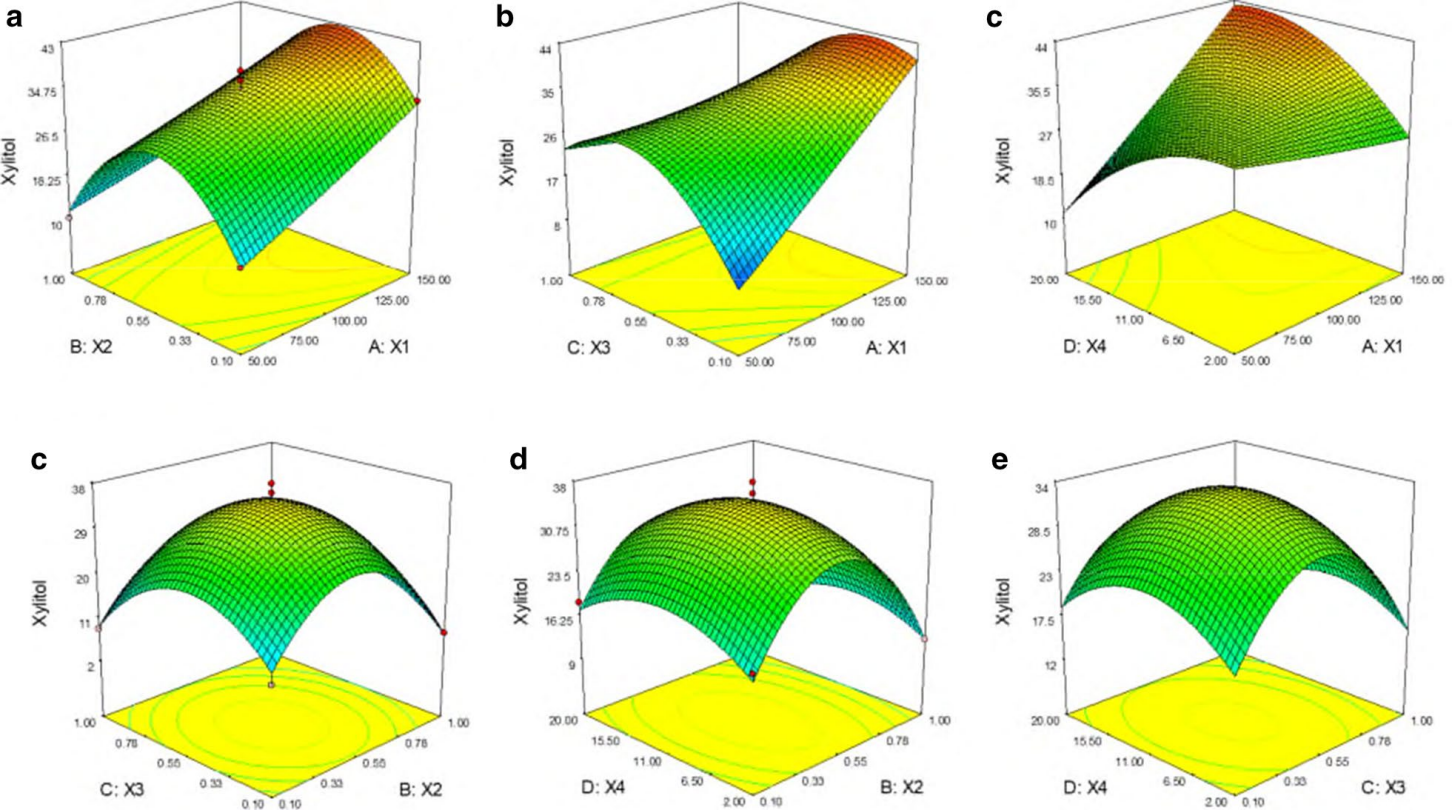
Three-dimensional response surface plot for xylitol production showing the interactive effects of **a** xylose (X1) and ammonium sulphate (X2), **b** xylose (X1) and KH_2_PO_4_ (X3), **c** xylose (X1) and yeast extract (X4), **d** ammonium sulphate (X2) and KH_2_PO_4_ (X3), **e** ammonium sulphate (X2) and yeast extract (X4), **f** KH2PO4 (X3) and yeast extract (X4) with the remaining factors kept constant at the middle level of the central composite experimental design

The tested variables well explained the production of the xylitol with the help of 3D surface plots. The plot was so built against any two independent variables that the response (xylitol titer) was plotted on *z*-axis while maintaining other variables at their mid-levels, as shown in the Fig. 5a–f. It is evidenced from the graph that there is a direct correlation between xylose concentration and xylitol production. There is a steep rise in xylitol production with an increasing initial concentration of xylose, leading to maximum xylitol production. Likewise, the same pattern is shown by yeast extract (Fig. 5c, e, f), where the optimum yeast extract concentration showed an improved production of xylitol. From these graphs, it can be noted that maximum xylitol production was achieved with a mid-level concentration of xylose and a higher concentration of yeast extract_._ On the contrary, at a mid concentration of ammonium sulfate (Fig. 5a, d, e) and KH_2_PO_4_ (Fig. 5b, d, f) turned out to be more helpful in increasing the xylitol production. Previously Ling et al. adapted central composite design (CCD) for enhancing xylitol production using *Candida tropicalis* HDY-02 [35]. They found that medium components such as KH_2_PO_4_, yeast extract, (NH_4_)_2_SO_4_ and MgSO_4_·7H_2_O had a significant effect on xylitol production. Likewise, Yewale et al. performed Plackett–Burman (PB) design followed by CCD and reported components such as KH_2_PO_4_, yeast extract and xylose are the critical factors for xylitol production [36]. These studies indicate the importance of statistical method to identify the vital components affecting growth and product formation.

**Fig. 6.**
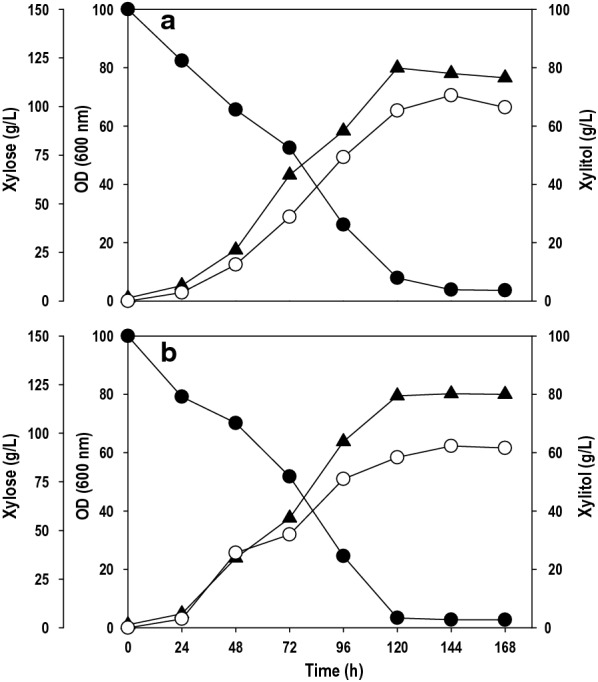
Shake flask cultivation of *P. fermentans *using optimized media on; **a** pure xylose and **b** xylose rich hemicellulosic pre-hydrolysate from sugarcane bagasse. Symbols: filled circle (xylose), filled triangle (OD_600_) and empty circle (xylitol). The graphs represent the average values of triplicate experiments performed with less than 10% S.D

The validation experiment was performed at the value obtained by Derringer’s desired function methodology shown in Additional file 1: Table S2. The target value was set for 50 g/L, and the desirable value was found to be 0.99, indicating higher probability to achieve the set target value. The optimal batch experiments were performed in two different sets in shake flask: with pure xylose (Fig. 6a) and xylose rich hemicellulosic pre-hydrolysate (Fig. 6b). The high initial xylose level of 150 g/L caused a lag phase of 24 h. After 24 h, xylose uptake was significantly improved, growth was picked up, and cells entered the log phase. A significant r fraction of the xylose was depleted between 120–144 h time intervals where a residual xylose concentration left was ~ 5.0 g/L. The cell growth enhanced linearly and nearly became steady after 120 h and reached ~ 80 (OD_600_). The results show that the cell growth was unaffected by inhibitors such as acetic acid and furfural present in the pre-hydrolysate. With pure xylose as a carbon source, the *P. fermentans* was able to convert 48.9% of xylose to xylitol with the titer of 70.5 g/L. In the case of pre-hydrolysate, the strain was able to accumulate 62.3 g/L of xylitol with the conversion yield of 42.7%. The optimized medium composition positively affected cellular growth and xylose assimilation, which eventually boosted xylitol synthesis. The concentration of xylitol produced is far superior to the results obtained by Huang et al. [19]; however, yields were lower. They used non-detoxified xylose rich rice straw and SCB hydrolysate for xylitol production by *Candida tropicalis* JH030. The yeast accumulated ~ 32.0 g/L with a conversion yield of 0.71 g/g from SCB while xylitol titer and yield achieved with rice straw were 12.5 g/L and 0.51 g/g, respectively. Similarly, Rodrigues et al. [37] obtained 36.3 g/L of xylitol with a yield of 0.63 g/g by *Candida guilliermondii* FTI 20037 using SCB hemicellulosic hydrolysate as a crude renewable source.

### Batch cultivation in bioreactor

**Fig. 7.**
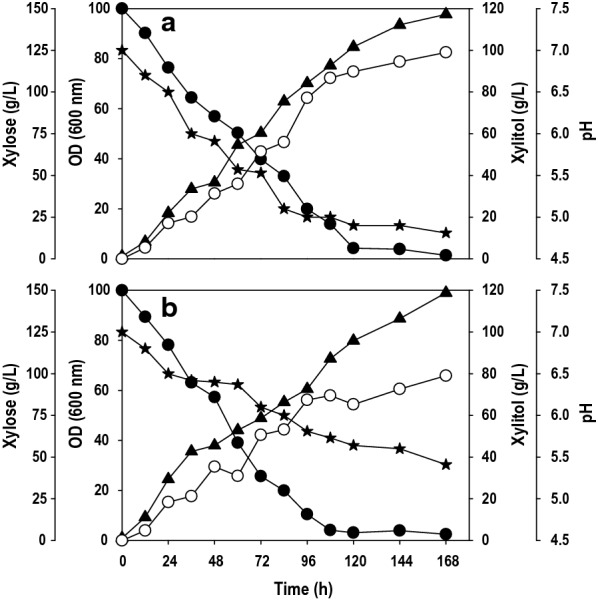
Batch kinetics of substrate assimilation, cell growth, pH and xylitol formation by *P. fermentans* in bioreactor on; **a** pure xylose and **b** xylose rich hemicellulosic pre-hydrolysate from sugarcane bagasse. Symbols: filled circle (xylose), filled triangle (OD_600_), empty circle (xylitol) and filled star (pH). The graphs represent the average values of triplicate experiments performed with less than 10% S.D

The data was scaled up from shake flask to bioreactor, and the batch experiments were performed in 2.5 L bench-scale bioreactor with 1 L working volume using pure xylose and pre-hydrolysate. The process parameters were the same as shake flask validation studies expect the aeration maintained at 1.0 L/min. The time course profiles for the fermentation are shown in Fig. 7. Xylose uptake, xylitol titer and yield were significantly improved from shake flask to bioreactor cultivation, and the xylose utilization was more than 95% in both the cases. The lag phase was reduced prominently in comparison to shake flask cultivation. The xylose underwent fast depletion, and the maximum cell OD_600_ of 98 was achieved at 168 h with pure xylose, which was significantly higher than obtained in shake flask studies. The highest amount of xylitol recorded was 98.9 g/L of xylitol with the yield of 0.67 g/g at 168 h time interval (Fig. 7a). The fermentation profile of *P. fermentans* with pre-hydrolysate is shown in Fig. 7b. The maximum cell OD_600_ achieved with pre-hydrolysate was 99, which is quite similar to growth obtained with pure xylose. However, the xylitol production (79 g/L) and yield (0.54 g/g) achieved were substantially lower than with pure xylose. Such improvement in xylitol production could be due to the better mixing effects and consequently improved mass transfer in the bioreactor. More particularly, the role of controlled aeration in a bioreactor cannot be overlooked as it directly affects the oxygen transfer rate, a key parameter affecting xylitol production as established earlier [13, 15, 31]. According to several literature reports, limited oxygen levels are favourable for xylitol accumulation, possibly due to elevated levels of XR and therefore, it is essential to restrict oxygen supply to maximize the biosynthesis of xylitol [38–40]. In the current study, the air was supplied at 1.0 vvm without control of dissolved oxygen (DO) level. After 24–48 h of cultivation, there was a significant drop in DO levels and remained below 5% during the rest of fermentation. The high rate of biomass formation resulted in rapid oxygen consumption and limited oxygen availability, boosting xylitol production.

For the cost-effective industrial production, it is desirable to have excellent tolerance against pH fluctuations and high substrate concentration by microbial strain. The pH declined in both the cases as fermentation progressed, and the final pH at the end of 168 h was 4.8 and 5.4 with pure xylose and pre-hydrolysate, respectively. The *P. fermentans* displayed excellent pH tolerance without compromising the xylitol production, which is an added advantage for the industrial production of xylitol. After optimizing media, when the xylose concentration was increased from 60 to 150 g/L, the yeast amassed an excellent level of xylitol from pure as well as crude xylose, and the yield was unaffected. Most of the xylitol producing yeasts displayed higher production when the initial xylose concentration was maintained between 100 to 200 g/L, and our results are in congruence with previous reports [31, 40]. The difference in the xylitol production with pure xylose and pre-hydrolysate could be attributed to the presence of known as well as unknown/undetected inhibitors, which negatively affected the performance.

In the last decade, a variety of wastes streams rich in xylose have been investigated for xylitol production. Table 3 compares the xylitol production achieved in the current study with other reports using pure xylose and xylose rich hemicellulosic hydrolysates with/without co-substrate for xylitol production. The range of xylitol titer and yield obtained in these studies broadly varies from 12.5 to 172.4 g/L and 0.51 to 1.0 g/g depending on feedstock/carbon source, inhibitor composition and/or detoxification method. The results obtained in current work are comparable or even better than data available in state of the art, making it competitive. It would be advantageous to manufacture xylitol from crude xylose without detoxification [19]. Usually, the xylitol yields with non-detoxified hemicellulosic hydrolysate are inferior in comparison to synthetic medium containing pure xylose. Therefore, the majority of the reports have made use of detoxified pre-hydrolysate during xylitol production, for improving the fermentation potential of microbes. However, in the current study, the high xylitol titer (79.0 g/L) and yield (0.53 g/g) were obtained with SCB pre-hydrolysate without detoxification, affirming the immense potential of *P. fermentans*.

**Table 3:** Comparison of xylitol production by different microorganisms

Microorganism	Feedstock	Detoxification	Xylitol titer (g/L)	Xylitol yield (g/g)	References
*Debaryomyces hansenii*	Pure xylose and glycerol	–	53.7	0.98	[25]
*Yarrowia lipolytica*	Pure xylose and glycerol	–	53.2	0.97	[44]
*Candida tropicalis* BSXDH-3	Pure xylose, glycerol and glucose	–	48.6	0.98	[46]
*Debaryomyces hansenii*	Pure xylose	–	68.6	0.76	[47]
*E. coli*	Pure xylose and glucose	–	172.4	–	[48]
*Kluyveromyces marxianus *YZJ015	Pure xylose	–	71.5	0.89	[50]
*P. fermentans*	Pure xylose	–	*98.9*	*0.67*	This study
*Candida tropicalis* JH030	Sugarcane bagasse	Non-detoxified	12.5	0.51	[19]
*Candida tropicalis* JH030	Rice straw	Non-detoxified	~ 32	0.71	[19]
*Candida tropicalis* W103	Corncob	Detoxified	68.4	0.70	[28]
*Candida athensensis* SB18	Horticultural waste	Detoxified	100.1	0.81	[34]
*Candida guilliermondii* FTI 20037	Sugarcane bagasse	Detoxified	36.3	0.64	[37]
*Debaryomyces hansenii*	Sugarcane bagasse	Detoxified	13.8	0.69	[47]
*E. coli*	Corncob and pure glucose	Detoxified	150.0	> 0.95	[48]
*Pichia stipitis*	Corn stover	Detoxified	12.5	0.61	[49]
*P. fermentans*	Sugarcane bagasse	Non-detoxified	*79.0*	*0.54*	This study

## Conclusions

The work describes the xylose assimilation and xylitol production capability of the newly isolated yeast strain *P. fermentans* from food waste. The strain was able to accumulate high levels of xylitol even at high concentration of pure as well as crude xylose, competitive with previous work. About 79.0 g/L xylitol with a conversion yield of 54% was obtained from xylose rich hemicellulosic pre-hydrolysate using *P. fermentans*. The advantageous feature of the strain is that it was able to synthesize xylitol from hydrolysate without detoxification and lack of detoxification requirements can make the process more economical. Unlike other hyper-producer of xylitol, *P. fermentans* is a safe organism and well-known for producing flavour and aroma metabolites with applications in the food and beverage industries. The work can also contribute towards establishing profitable lignocellulosic biorefineries in the UK through the valorisation of abundantly available xylose-rich waste streams such as stem wood, scots pine, Norway spruce etc. Low volumetric productivity and the substantial amount of complex nitrogen sources used in the current work are the bottlenecks for bulk production of xylitol and needs to be taken care. *P. fermentans* could be used as a starting point, and the commercial feasibility for xylitol production could be achieved by the application of metabolic engineering approaches and bioprocess optimization.

## Methods

### Materials used in this study

All the chemicals used in this study were of analytical grade and purchased from Sigma Aldrich (USA). The molecular biology reagents were procured from New England Biolabs (USA) and NBS Biologicals (UK). The xylose rich pre-hydrolysate obtained after hydrothermal pretreatment of SCB was kindly provided by our industrial partner Nova Pangaea Technologies, (https://www.novapangaea.com), Redcar, UK. The composition of the concentrated hemicellulosic hydrolysate was as follows: xylose, 500.0 g/L; glucose, 50.0 g/L; arabinose, 37.5 g/L; acetic acid, 55.0 g/L; furfural < 1.0 g/L.

### Isolation and maintenance of xylose assimilating yeast

The xylose assimilating yeast used in the present study was isolated from mixed food waste, collected from Cranfield Student Association (CSA) (52° 04′ 20.4" N 0° 37′ 45.0" W) at Cranfield University, UK. The morphological characteristics were analyzed with microscopic observation. The isolated yeast showed an ellipsoid structure having 3–4 µm diameter (Fig. 1a). Further, 26 s rRNA and ITS (internal transcribing spacers) sequence of isolated yeast were aligned with sequences available in the NCBI database using Clustal W and Mega 10.0 software. The alignment results showed that isolated yeast strain shared 99% homology with *Pichia fermentans*. Phylogenetic trees were constructed based on 26S rRNA sequence of the isolated strain using the Neighbor-Joining method; further, the distance between the evolutionary species were determined according to Kimura 2-model [41] (Fig. 1b).

It was routinely maintained on modified yeast extract-xylose-malt extract (YXM) agar containing 3.0 g/L yeast extract, 3.0 g/L malt extract, 5.0 g/L peptone, 10.0 g/L xylose, 20.0 g/L agar. Bacterial contamination was prevented by adding 10.0 µg/mL chloramphenicol to the said medium and adjusting initial pH to 3.5. The yeast was regularly sub-cultured every 2 weeks and plates were stored at 4 °C in the refrigerator.

### Shake flask cultivation of *P. fermentans* for xylitol production

Shake flask studies were conducted to investigate the xylitol production potential of *P. fermentans*. Two different strategies were adopted to assess the ability of the strain for xylitol production, one being the use of single substrate or pure xylose and other being the use of glucose (10 g/L) as co-substrate with xylose. The medium used for preparing seed culture and submerged xylitol fermentation was according to Cheng et al. [42] with slight modification, having the following composition: 10.0 g/L yeast extract, 20.0 g/L peptone, 30.0 g/L xylose, 0.5 g/L KH_2_PO_4_, 0.5 g/L (NH_4_)_2_SO_4_, and 0.5 g/L MgSO_4_. The seed culture was grown in a 100 mL Erlenmeyer flask containing 25 mL of the above-mentioned medium using a single colony of *P. fermentans*. The final pH of the medium before sterilization was adjusted to 7.0. Cultivation was carried out for 24 h at 30 °C on a rotary shaker with an agitation speed of 250 rpm. The submerged cultivations were carried out in 500 mL Erlenmeyer flasks containing 100 mL working volume. The flasks were inoculated with fresh inoculum at OD_600_ of 1.0 and kept at 30 °C under constant shaking at 250 rpm on a rotary shaker (Excella 24, New Brunswick).

### Random mutagenesis using ethyl methanesulphonate (EMS)

The chemical mutagenesis was performed according to the protocol by [43]. A single colony of *P. fermentans* was grown overnight in 5 mL of YPX medium at 30 °C on a rotary shaker with an agitation speed of 250 rpm and then centrifuged. The collected cell mass was washed thrice with sterile distilled water and suspended in 10 mL of phosphate buffer (0.1 M, pH 7.0). The sample concentration was adjusted to obtain the cell count of ~ 2 × 10^8^ cells/mL. One mL of the cell solution was centrifuged for 30 s at 20,000×*g*. The cell pellet was suspended in 1 mL of phosphate buffer (0.1 M, pH 7.0) in a 15 mL sterile centrifuge tube and 50 µL of 20 mM EMS was added to the cell suspension. Later the cell suspension was vortexed and incubated  for different intervals of time at 30 °C. The reaction was stopped by adding 8.0 mL of 5% sterile sodium thiosulfate to 0.2 mL of mutated culture. Finally, 0.1 mL of the culture was plated on YPX agar medium at pH 7.0 and 28 °C until colonies were formed.

### Measurement of enzymatic activities

The enzymatic activities of xylose reductase (XR) and xylitol dehydrogenase (XDH) were measured according to the protocol as described earlier in Prabhu et al. [44].

### Media optimization to maximize xylitol production using Box Behnken design

Media optimization to maximize xylitol production was carried out using Box Behnken Design (BBD) as described in Prabhu et al. [45]. The medium components chosen for optimization were xylose, ammonium sulphate (NH_4_)_2_SO_4_), potassium dihydrogen phosphate (KH_2_PO_4_) and yeast extract. The levels of each variable are shown in Additional file 1: Table S1. The optimal levels were predicted with the aid of second-order polynomial.

### Batch cultivation in bioreactor

The batch experiments were performed in a 2.5 L bioreactor (Electrolab Bioreactors, UK) with 1.0 L working volume. The inoculum was prepared using optimized media, and the optimum values of media components were as follows (g/L): xylose, 150.0; KH_2_PO_4_, 0.30; (NH_4_)_2_SO_4_, 0.46; yeast extract, 18.36. The starting pH was 7.0 and not controlled during the fermentation. The temperature and agitation speed were maintained  at 30 °C and 250 rpm, respectively, while the aeration rate was maintained at 1.0 L/min.

### Analytical methods

The samples were withdrawn periodically and analysed for OD, pH, residual glucose, xylose and xylitol. Cell growth was quantified by measuring the optical density at 600 nm wavelength in a 1 mm-path-length cuvette using a double beam spectrophotometer (Jenway 6310, UK). The concentrations of glucose, xylose and xylitol were measured by HPLC (Agilent Technologies 1200 series, USA) as described in Prabhu et al. [44]. All measurements were conducted in triplicates and values were averaged. The standard deviation was not more than 10%.

## Highlights

- Isolation of xylitol accumulating yeast identified as *Pichia fermentans*.
- Random mutagenesis caused ~ 26 and 51% improvement in xylitol titer and yield, respectively.
- Xylitol titre and yield achieved with pure xylose were 98.9 g/L and 0.67 g/g, respectively.
- Xylitol accumulated using xylose rich pre-hydrolysate from sugarcane bagasse was 79.0 g/L with an overall yield of 0.54 g/g.

## Supplementary Information

**Additional file 1.** **Table S1.** Coded values of independent variables for Box benkhen design. **Table S2.** Derringer’s desired function for the optimum value.

## Data Availability

All data generated or analyzed during this study are included in the manuscript.
