# Supplementary material for: Enhanced xylitol production using non-detoxified xylose rich pre-hydrolysate from sugarcane bagasse by newly isolated Pichia fermentans
Source: Biotechnol Biofuels. 2020 Dec 29;13:209. doi: 10.1186/s13068-020-01845-2 (PMC7772924; doi:10.1186/s13068-020-01845-2)
Supplement: Supplementary file 1 — Additional file 1. Table S1. Coded values of independent variables for Box benkhen design. Table S2. Derringer’s desired function for the optimum value. [file 13068_2020_1845_MOESM1_ESM.docx]

**Table S1.** **Coded values of independent variables for Box benkhen design**

| Independent variable |  | Coded value | | |
| --- | --- | --- | --- | --- |
|  | Symbol code | -1 | 0 | 1 |
| Xylose (g/L) | X_1_ | 50 | 100 | 150 |
| Ammonium sulphate (g/L) | X_2_ | 0.1 | 0.55 | 1 |
| KH_2_PO_4_ (g/L) | X_3_ | 0.1 | 0.55 | 1 |
| Yeast extract (g/L) | X_4_ | 2 | 11 | 20 |

**Table S2:** Derringer’s desired function for the optimum value

| Global Solution |  | Predicted Responses | |
| --- | --- | --- | --- |
| Xylose | 150 | Xylitol | 49.63 |
| Ammonium sulphate | 0.46 | Desirability | 0.99 |
| KH_2_PO_4_ | 0.3 |  |  |
| Yeast extract | 18.36 |  |  |
